# *Ab Initio* prediction of mycobacteriophages protein structure and function

**DOI:** 10.1186/1471-2105-14-S17-A10

**Published:** 2013-10-22

**Authors:** Chiraag D Kapadia, Claire A Rinehart

**Affiliations:** 1 Department of Biology, Western Kentucky University, Bowling Green, KY, 42101, USA

## Background

*Mycobacterium smegmatis* is a soil bacterium. Over 448 mycobacteriophages specific for *M. smegmatis* have been sequenced and grouped into clusters of related genomes based on the similarity of their products and genome organization. Only 20% of mycobacteriophage genes have known function, as predicted by protein sequence level alignments [1].

## Materials and methods

Genes that are grouped together using BLAST at the protein sequence level have been assembled into loose groupings called phams [2]. The phagesdb.org/phams database contains the protein sequences organized by phams. From these data we used *ab initio* folding, using I-TASSER [3], to predict the structure of multiple phams across numerous mycobacteriophage clusters. Predicted models were grouped into structural families based upon RMSD scores from pairwise comparisons. Models from two structural families per pham were submitted to COFACTOR [4], which finds the best structural homologies to proteins in the PDB library and returns the matching structures along with GO terms, EC numbers and active site information.

## Results

**Figure 1:**
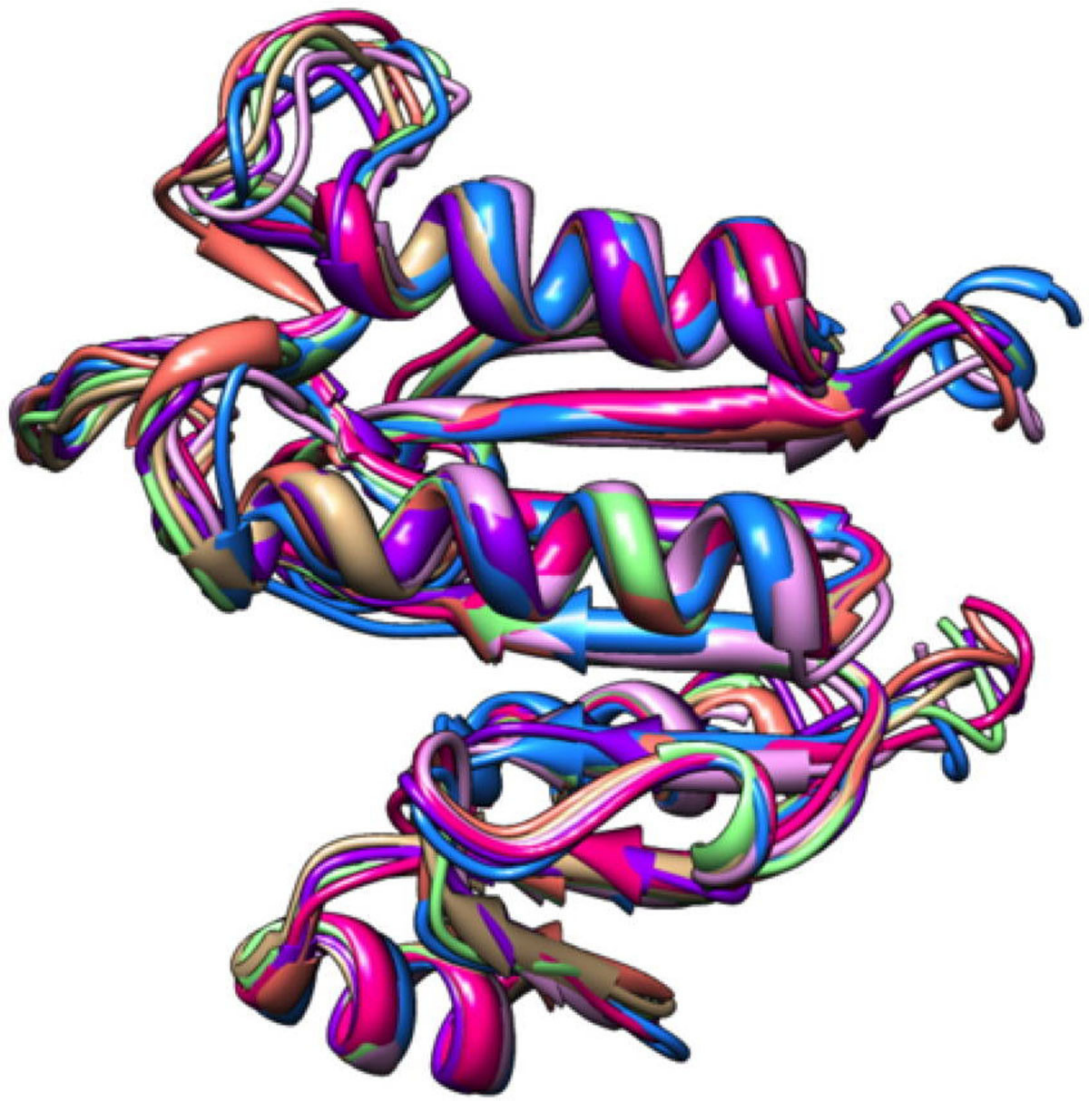
Seven predicted pham 6714 structures superimposed into a single model matching methyltransferase structures. PDB files were rendered using UCSF Chimera (http://www.cgl.ucsf.edu/chimera/). Proteins include MeeZee gp 78, JN243856.1; Backyardigan gp 74, JF704093.1; and ShiLan gp 70, JN020143.1

**Figure 2:**
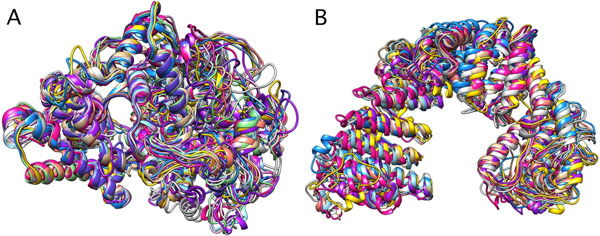
Ten predicted pham 2789 structures superimposed in two possible models matching either a neutral endopeptidase (A) or an importin protein (B). PDB files were rendered using UCSF Chimera (http://www.cgl.ucsf.edu/chimera/). Proteins include Ava3 gp 250, JQ911768.1; Rizal gp 242, EU826467.1; and Pio gp 260, JN699013.1

Based on COFACTOR output, we were able to suggest functions for the genes in each respective pham examined. Two notable results: 1) pham 6714 is predicted to be a methyltransferase (Figure 1), and 2) pham 2789 is predicted to be either a neutral endopeptidase (Figure 2a) or an importin protein (Figure 2b). These predicted functions will need to be confirmed experimentally.
